# Effect of sexual transmission on the West Africa Ebola outbreak in 2014: a mathematical modelling study

**DOI:** 10.1038/s41598-018-38397-3

**Published:** 2019-02-07

**Authors:** Dongmei Luo, Rongjiong Zheng, Duolao Wang, Xueliang Zhang, Yi Yin, Kai Wang, Weiming Wang

**Affiliations:** 1 Department of Student Affairs, The 3rd Affiliated Teaching Hospital of Xinjiang Medical University (Affiliated Cancer Hospital), Urumqi, 830011 P. R. China; 2 Department of Infectious Diseases, The first Affiliated Hospital of Xinjiang Medical University, Urumqi, 830054 P. R. China; 3 Biostatistics Unit, Department of Clinical Sciences, Liverpool School of Tropical Medicine Pembroke Place, Liverpool, L3 5QA UK; 4 College of Medical Engineering and Technology, Xinjiang Medical University, Urumqi, 830011 P. R. China; 5 School of Mathematics Science, Huaiyin Normal University, Huaiyin, 223300 P. R. China

## Abstract

The outbreak of the Ebola virus has resulted in significant morbidity and mortality in the affected areas, and Ebola virus RNA has been found in the semen of the survivors after 9 months of symptom onset. However, the role that sexual transmission played in the transmission is not very clear. In this paper, we developed a compartmental model for Ebola virus disease (EVD) dynamics, which includes three different infectious routes: contact with the infectious, contact with dead bodies, and transmission by sexual behaviour with convalescent survivors. We fitted the model to daily cumulative cases from the first reported infected case to October 25, 2014 for the epidemic in Sierra Leone, Liberia and Guinea. The basic reproduction numbers in these countries were estimated as 1.6726 (95%CI:1.5922–1.7573), 1.8162 (95%CI:1.7660–1.8329) and 1.4873 (95%CI:1.4770–1.4990), respectively. We calculated the contribution of sexual transmission to the basic reproduction number *R*_0_ as 0.1155 (6.9%), 0.0236 (2.8%) and 0.0546 (3.7%) in Sierra Leone, Liberia and Guinea, respectively. Sensitivity analysis shows that the transmission rates caused by contacts with alive patients and sexual activities with convalescent patients have stronger impacts on the *R*_0_. These results suggest that isolating the infectious individuals and advising the recovery men to avoid sexual intercourse are efficient ways for the eradication of endemic EVD.

## Introduction

The Ebola virus, a member of the filoviridae virus family, caused an unprecedented outbreak in West Africa in 2014 which was counted as the largest Ebola epidemic ever observed. The disease, which spreads in humans and other mammalian populations, has an average death rate of about 70%^1,2^. The incubation period of Ebola can last from 2 to 21 days, usually 8–10 days^3^. There is not enough information indicating patients being infectious to another people at latency. After the incubation period, patients show sudden onset of fever, sore throat, muscle pain, and headaches. Then the illness becomes more acute with vomiting, diarrhea, body rash, tremors and in some cases, internal and external bleeding^4^.

Ebola is transmitted person-to-person primarily via direct contact with body fluids, secretions, tissue, contaminated surfaces and materials, from living, infected or recently deceased people^5^. The burial ceremonies and handling of dead bodies play a key role in transmission of the Ebola virus, because dead but not buried bodies can still transmit the virus. Family and community members exposed to the funerals present an exceptionally high risk for disease contamination^6,7^.

Recent reports suggest that the virus can persist in the semen of convalescent survivors for several months^8–17^, and as an instance, sexual transmission has been confirmed from studies shown that the semen of male survivor was positive by reverse transcription polymerase chain reaction (RT-PCR)^11^. On May 7th, 2015, the WHO recommended testing the semen from male Ebola survivors monthly, until their semen tests showed negative twice by RT-PCR^8^. Though the majorly affected countries Sierra Leone, Liberia and Guinea, are declared free of Ebola. Leaving more than 17,000 survivors, 3 cases of sexual transmission have been documented from these countries^8^. Sexual transmission of Ebola from convalescent survivors is a rare event, but researchers have warned that it should be considered in epidemiological models to understand the trajectory of an outbreak^16^.

A number of mathematical models have been developed to understand the transmission dynamics of Ebola and other infectious diseases outbreak from various aspects (see for instance^15,16,18–45^). Furthermore, several mathematical studies for the transmission dynamics only focus on the direct transmission or only consider sexual transmission: Lewnard *et al*. constructed a mathematical model with individuals divided into susceptible, latently infected, infectious, recovered and infectious people who have died from the disease^33^. They fitted the model to the reported cumulative Ebola cases and deaths in Montserrado County, Liberia, and assessed various intervention strategies in controlling the spread of Ebola virus in the country. In another study, Drake *et al*. carried out a multi-type branching process model and used time-dependent parameters to reflect increasing intensity of intervention efforts, incorporate the rates of hospitalization, exposure of healthcare workers, and secure burial^34^. Similarly, Barbarossa *et al*. developed a compartmental model including virus transmission in the community, at hospitals, and at funerals, and they derived a final epidemic size by forecasting the total number of cases during the outbreak^27^. In a recent study, Abbate *et al*. developed an SEIR compartmental model that includes sexual transmission from convalescent survivors^16^. Using the model, they evaluated the potential effect of sexual transmission on Ebola virus epidemiologically. Similarly, Vinson *et al*. presented a mathematical model that comprised the direct contact and sexual transmission modes^15^. Mathematical analysis showed that reducing sexual transmission could play a significant role in eradication of the disease. Gao *et al*.^45^ presented a mathematical model to investigate the impact of mosquito-borne and sexual transmission on spread and control of ZIKV and used the model to fit the ZIKV data in Brazil, Colombia, and El Salvador.

Inspired by these previous literatures, in this paper, without aiming to make a predictive model but rather to understand how the epidemic was affected by various transmission routes which include contact with infections, contact with dead bodies, and having sex with convalescent survivors, we develop a mathematical model which considers the three main features of the disease transmission and fit to the reported cumulative case data of infected cases in Sierra Leone, Liberia, and Guinea.

The article is organized as follows. In Section 2, we introduce the EVD model and present the expression of the basic reproduction number, and introduce the data sources. The model fitting and contribution of related component to the basic reproduction number *R*_0_ in Sierra Leone, Liberia and Guinea are carried out in Section 3. In Section 4, we give a brief summary and some discussions.

## Methods and Materials

### Model Formulation

**Figure 1:**
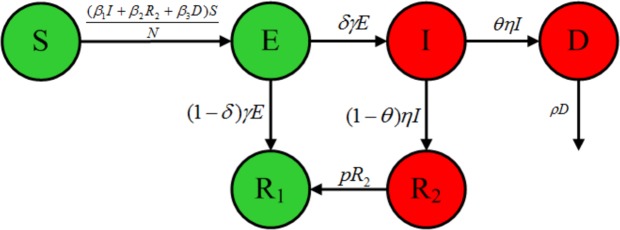
Compartmental diagram of the Ebola transmission model.

To study the transmission of Ebola in West Africa we developed a EVD model, where the population is divided into six groups: the susceptible individuals *S*, exposed individuals *E*, symptomatic and infectious individuals *I*, deceased infected individuals *D*, convalescent survivors who maintain active Ebola virus replication *R*_2_, and fully recovered and immune people *R*_1_, such that the total population is *N* = *S* + *E* + *I* + *R*_1_ + *R*_2_ + *D*. According to the natural history of EVD, some people have an innate immunity to Ebola and the diseases may not be fatal to all^46,47^, so the exposed individuals may go directly to the recovery class *R*_1_. Because we considered a relatively short time frame (<1 year), we assumed for simplicity that the total population is constant and hence we omit population births and deaths when modeling the outbreak. Additionally, we assumed that people who recover from the Ebola infection develop antibodies that last for at least 10 years, so recovered individuals will not move to compartment *S* in a short time. A flow of individuals from one class to another is depicted in Fig. 1. The transmission process of Ebola virus is then described by the following a system of six ordinary differential equations:

1 $$\{\begin{array}{rcl}\frac{{\rm{d}}S}{{\rm{d}}t} & = & -({\beta }_{1}I+{\beta }_{2}{R}_{2}+{\beta }_{3}D)\frac{S}{N},\\ \frac{{\rm{d}}E}{{\rm{d}}t} & = & ({\beta }_{1}I+{\beta }_{2}{R}_{2}+{\beta }_{3}D)\frac{S}{N}-\gamma E,\\ \frac{{\rm{d}}{R}_{1}}{{\rm{d}}t} & = & \mathrm{(1}-\delta )\gamma E+p{R}_{2},\\ \frac{dI}{dt} & = & \delta \gamma E-\eta I,\\ \frac{d{R}_{2}}{dt} & = & \mathrm{(1}-\theta )\eta I-p{R}_{2},\\ \frac{dD}{dt} & = & \theta \eta I-\rho D.\end{array}$$

We assumed susceptible individuals *S* could be infected via three paths: person-to-person transmission *β*_1_, sex with convalescent survivors *β*_2_ and deceased individuals *β*_3_. 1/*γ* and 1/*η* represent the average durations of incubation and infection, respectively. *δ* represents the proportion of exposed *E* progress to the infectious *I*, *θ* is the case fatality rate. Deceased individuals can be buried directly during funerals at rate *ρ*. The average duration for convalescent patients to recover is assumed as 1/p.

### Basic reproduction number

The basic reproductive number *R*_0_ is the average number of secondary cases caused by an infectious individual during his/her entire infectious period. To calculate *R*_0_, we used the next generation matrix method^48,49^, where

2 $$ {\mathcal F} =(\begin{array}{c}({\beta }_{1}I+{\beta }_{2}{R}_{2}+{\beta }_{3}D)\frac{S}{N}\\ 0\\ 0\\ 0\end{array}),\,{\mathscr{V}}=(\begin{array}{c}\gamma E\\ \eta I-\delta \gamma E\\ P{R}_{2}-\mathrm{(1}-\theta )\theta I\\ \rho D-\theta \eta I\end{array}).$$

Then

3 $$F=(\begin{array}{llll}0 & {\beta }_{1} & {\beta }_{2} & {\beta }_{3}\\ 0 & 0 & 0 & 0\\ 0 & 0 & 0 & 0\\ 0 & 0 & 0 & 0\end{array}),\,V=(\begin{array}{cccc}\gamma  & 0 & 0 & 0\\ -\delta \gamma  & \eta  & 0 & 0\\ 0 & -\mathrm{(1}-\theta )\eta  & p & 0\\ 0 & -\theta \eta  & 0 & \rho \end{array}).$$

*V* ^−1^ is given by

4 $${V}^{-1}=(\begin{array}{llll}\frac{1}{\gamma } & 0 & 0 & 0\\ \frac{\delta }{\eta } & \frac{1}{\eta } & 0 & 0\\ \frac{\delta \mathrm{(1}-\theta )}{p} & \frac{1-\theta }{p} & \frac{1}{p} & 0\\ \frac{\delta \theta }{\rho } & \frac{\theta }{\rho } & 0 & \frac{1}{\rho }\end{array})$$

Thus

5 $$F{V}^{-1}=(\begin{array}{cccc}\frac{{\beta }_{1}\delta }{\eta }+\frac{{\beta }_{2}\delta \mathrm{(1}-\theta )}{p}+\frac{{\beta }_{3}\delta \theta }{\rho } & \frac{{\beta }_{1}}{\eta }+\frac{{\beta }_{3}\theta }{\rho }+\frac{{\beta }_{2}\mathrm{(1}-\theta )}{p} & \frac{{\beta }_{2}}{p} & \frac{{\beta }_{3}}{\rho }\\ 0 & 0 & 0 & 0\\ 0 & 0 & 0 & 0\\ 0 & 0 & 0 & 0\end{array}).$$

The reproduction number is given by *ρ*(*FV*^−1^), and

$${R}_{0}=\mathop{\underbrace{\frac{{\beta }_{1}\delta }{\eta }}}\limits_{{R}_{I}}+\mathop{\underbrace{\frac{{\beta }_{2}\delta \mathrm{(1}-\theta )}{p}}}\limits_{{R}_{S}}+\mathop{\underbrace{\frac{{\beta }_{3}\delta \theta }{\rho }}}\limits_{{R}_{D}}.$$

In order to interpret *R*_0_ in terms of the dynamics of Ebola spread, the basic reproduction number for the system breaks down to three components: the new infection induced by contacts with infectious individuals *R*_*I*_, sexual activities with convalescent survivors *R*_*S*_, and contacts with dead bodies *R*_*D*_.

### Data and Parameter Estimation

**Figure 2:**
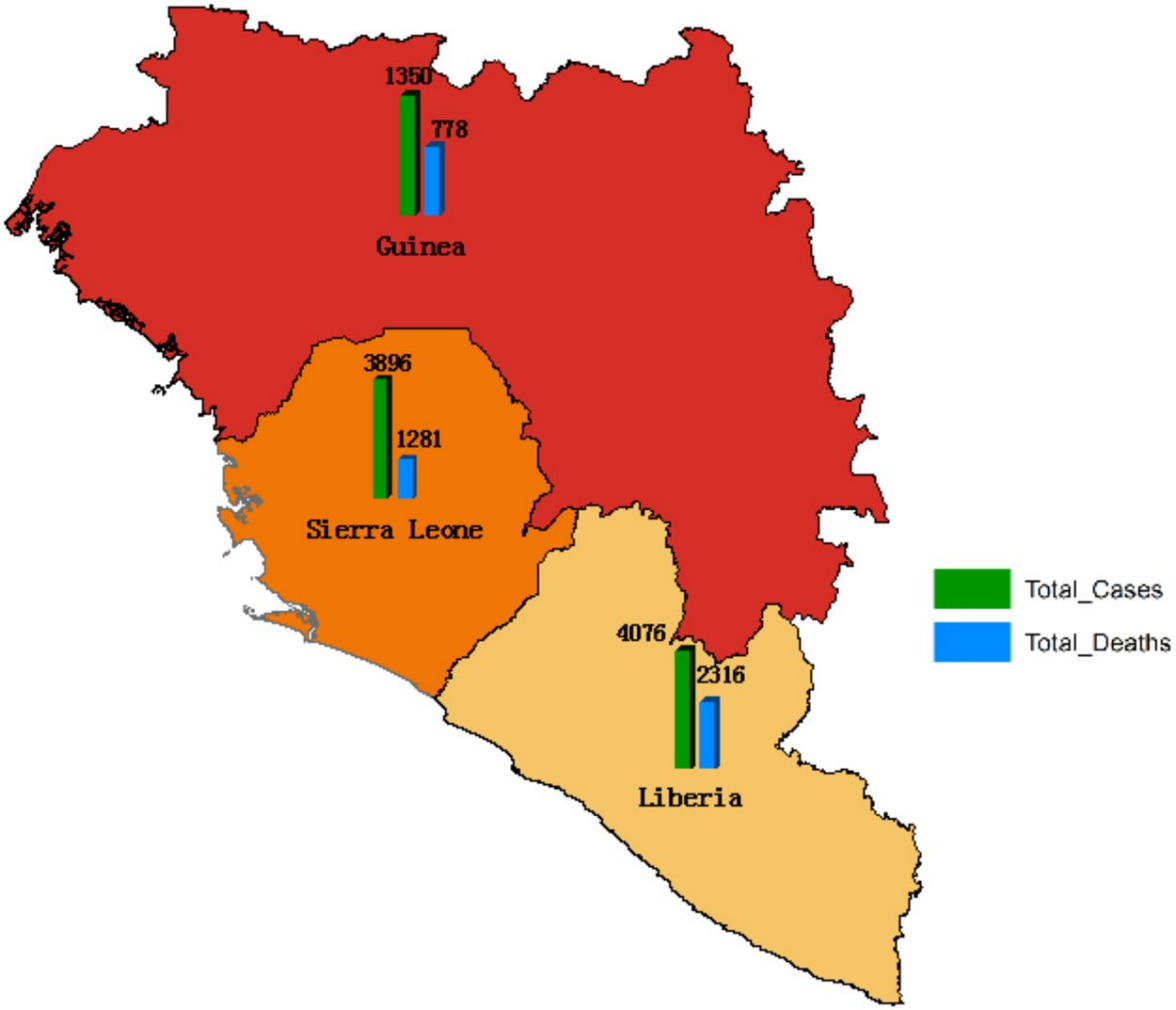
The Ebola cumulative cases during the outbreaks in Sierra Leone, Liberia and Guinea, up to October 25th, 2014.

We obtained data of the daily cumulative cases which is publicly available online from the Centers for Disease Control and Prevention^50^, which allows the user to obtain information about the daily number of Ebola cases (confirmed, probable and deaths) in Guinea, Sierra Leone, Liberia. We used the start date for each country as when the first infectious case was reported. According to the available data provided by the CDC, the first cases reported were on May 27th, 2014 for Sierra Leone, March 27th, 2014 for Liberia and March 25th, 2014 for Guinea. As of October 25, 2014, the cumulative cases and deaths in each country are shown in Fig. 2. We use the cumulative cases up to October 25, 2014 to fit model (1).

The values of parameters for model (1) are summarized in Table 1. To parameterize our model, we adopted the epidemiological parameters from literatures. For the total population size, *N*, we used a total population of 7,079,162 for Sierra Leone, 4,390,737 for Liberia, and 11,805,509 for Guinea, as provided by the World Bank^51^. In view of the reported cases from the EVD outbreak in West Africa in 2014, we chose the mean incubation period 1/*γ* as 9.31 days and the mean infectious duration 1/*η* as 7.41 days. The average duration from death to burial 1/*ρ* is assumed as 2 days. The disease-related death rations, *θ*, according to previous studies^52^, were the case fatality rates 0.690 for Sierra Leone, 0.723 for Liberia, and 0.707 for Guinea. We assume that Sierra Leone, Liberia and Guinea share the same values of *γ*, *η*, and *ρ* [see Table 1].

**Table 1:** Parameter descriptions and estimations.

Parameter	Sierra Leone	Liberia	Guinea	Description	Source
*N*	7,079,162	4,390,737	11,805,509	Total human population	^51^
1/*γ*	9.31 days	9.31 days	9.31 days	Mean incubation period	^35^
1/*η*	7.41 days	7.41 days	7.41 days	Mean infection period	^35^
*θ*	0.690	0.723	0.707	Case fatality rate	^3^
1/*ρ*	2 days	2 days	2 days	Mean time from death to burial	^16^
1/*p*	87.35 days	87.35 days	87.35 days	Mean time to convalescent survivors recover completely	^52^
*β*_1_	0.1320 [0.1298, 0.1332]	0.1723 [0.1708, 0.1746]	0.1561 [0.1510, 0.1588]	transmission rate of contacting alive infections patients	[Fitted]
*β*_2_	0.0054 [0.0008, 0.0084]	0.0037 [0.0013, 0.0049]	0.0021 [0.0014, 0.0033]	Sexual transmission rate	[Fitted]
*β*_3_	0.4711 [0.4491, 0.4866]	0.5049 [0.4930, 0.5226]	0.2443 [0.2284, 0.2740]	transmission rate of contacting dead bodies	[Fitted]
*δ*	0.9511 [0.9219, 0.9697]	0.8639 [0.8525, 0.8733]	0.9584 [0.9353, 0.9682]	proportion of removed infection individuals	[Fitted]
*R*_0_	1.6726 [1.5922, 1.7573]	1.8162 [1.7660, 1.8329]	1.4873 [1.4770, 1.4990]	The basic reproduction number	[Calculated]
*R*_*I*_	0.9331 [0.9048, 0.9480]	1.1053[1.0895, 1.1161]	1.0890 [1.0473, 1.1174]	The infectious class’s contribution to *R*_0_	[Calculated]
*R*_*S*_	0.1155 [0.0066, 0.2343]	0.0236 [0.0860, 0.1034]	0.0546 [0.0335, 0.0853]	The convalescent class’s contribution to *R*_0_	[Calculated]
*R*_*D*_	0.6218 [0.6075, 0.6479]	0.6250 [0.6138, 0.6408]	0.3493 [0.3148, 0.3705]	The death class’s contribution to *R*_0_	[Calculated]

Although some parameters are not available, we can still make some reasonable assumptions. We used the value 87.35 for 1/*p* based on published values from the literature, and for all three countries.

We model cumulative cases as a Poisson-distributed random variable because the Poisson distribution describes the number of observed events in an interval of time. We calibrate the model by sampling from the posterior distribution of parameter vector *θ*|y = {*β*_1_, *β*_2_, *β*_3_, *δ*}|y, where vector y is derived from $\frac{{\rm{d}}}{{\rm{d}}{\rm{t}}}Y(t)=\delta \gamma E$ and *Y*(*t*) denotes the reported cumulative cases. We conduct sampling via Markov Chain Monte Carlo using a Metropolis-Hastings acceptance rule. The posterior density is

$${f}_{\Theta |{\rm{y}}}(\theta |{\rm{y}})=\prod _{T} {\mathcal L} (Y(t)|\theta ){f}_{\Theta }(\theta \mathrm{)}.$$

**Figure 3:**
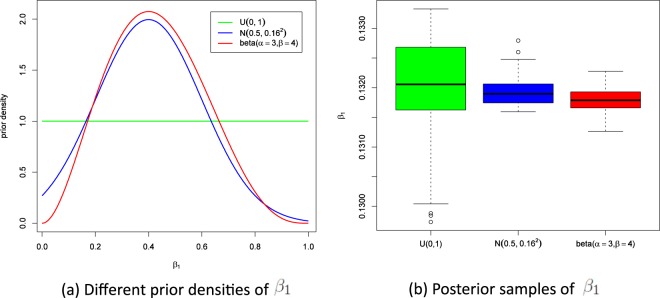
Prior densities and Posterior samples of *β*_1_.

The prior density *f*_Θ_(*θ*) is the joint probability of four univariate priors. We consider that *β*_1_, *β*_2_, *β*_3_, and *δ* are distributed according to ${\mathscr{U}}(0,\,1)$. The program was implemented in R version 3.3.1. We sampled from 30,000 MCMC iterations and discarded the first 10,000 samples as a burn-in period. On the basis of these 20,000 samples, the point estimates and 95% confidence intervals for the transmission coefficients were calculated, and the three kinds contribution to *R*_0_ can be calculated based on existing parameter values. The results are shown in Table 1. In order to check the influence of different prior distributions for estimated parameters, we choose ${\mathscr{U}}(0,\,1)$, ${\mathscr{N}}{(0.5,0.16}^{2})$ and beta(*α* = 3, *β* = 4) for prior distribution, respectively. The estimated values of *β*_1_ are 0.13204, 0.13189 and 0.13178 (See Fig. 3). This shows that the prior distributions have little influence on parameter *β*_1_. Similar results can be seen for parameters *β*_2_, *β*_3_, and *δ*.

## Result

### Model Fitting

**Figure 4:**
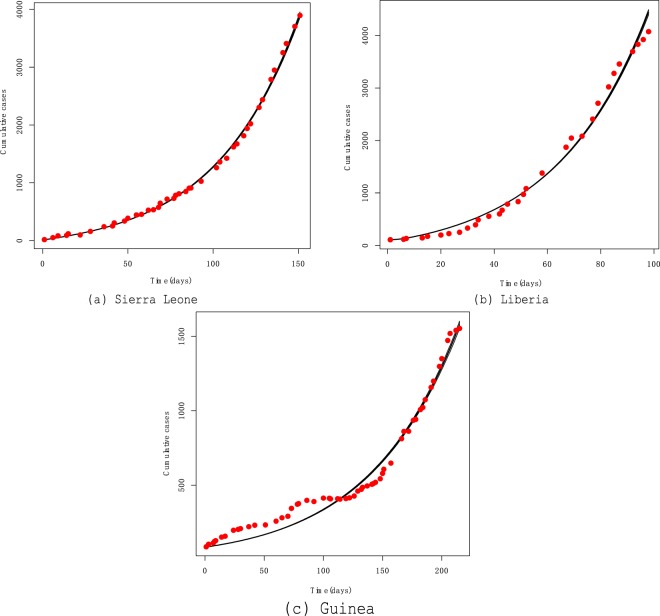
Fitting model to data in Sierra Leone, Liberia and Guinea as of October 25, 2014.

Model fitting was performed using MCMC method. In numerical experiments, the daily number of Ebola cases for each country is used to fit the cumulative number of infected cases. The model fits the cumulative reported cases in Sierra Leone, Liberia, and Guinea well generally (see Fig. 4). Figure 4 demonstrates that the fitting result of Sierra Leone is the best of these three countries, as Liberia and Guinea reported data with fluctuations, especially Guinea. In any case, the cumulative numbers of disease in October continues to increase in three countries. It should be noted that our model does not a consider any interventions, since, intervention efforts were escalated significantly after October 1, 2014^20^.

### Contribution of related component to *R*_0_

**Figure 5:**
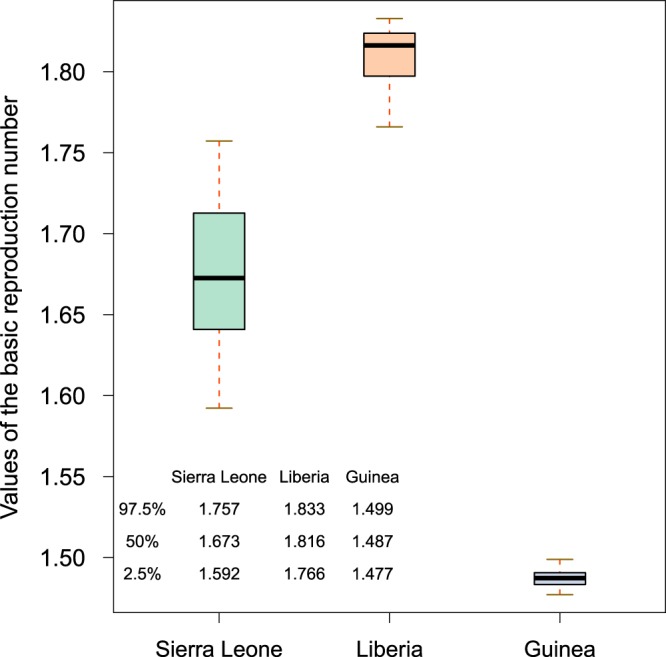
Boxplots of the basic reproduction numbers of Sierra Leone, Liberia and Guinea.

Based on the model and the estimated parameter values in Table 1, we calculated the basic reproduction number *R*_0_ and 95% confidence interval in Sierra Leone, Liberia and Guinea as 1.6726 (95%CI:1.5922–1.7573), 1.8162 (95%CI:1.76601–1.8329) and 1.4873 (95%CI:1.4770–1.4990), respectively (see Fig. 5). The simulated results indicate that Liberia is the most seriously affected area in three countries at that time. In contrast, our *R*_0_ agrees with previous works summarized in Table 2. For instance, the WHO Ebola Response Team^52^ estimated the basic reproduction number of Ebola is in the range of [1.79, 2.26] in Sierra Leone, [1.72, 1.94] in Liberia and [1.44, 2.01] in Guinea. Similarly, Shen calculated the *R*_0_ in Sierra Leone, Liberia and Guinea, as 1.6093, 1.7994 and 1.2552, respectively^26^.

**Table 2:** Comparison with previously published estimates *R*_0_.

Location	*R*_0_	95% Confidence Interval	Reference
Sierra Leone	1.18	—	^54^
	1.6093	[1.5609, 1.6577]	^26^
	1.6726	[1.5922, 1.7573]	Obtained here
	1.77	—	^18^
	2.02	[1.79, 2.26]	^52^
	2.53	[2.41, 2.67]	^55^
Liberia	1.22	—	^54^
	1.59	[1.57, 1.60]	^55^
	1.7994	[1.7655, 1.8333]	^26^
	1.8162	[1.7660, 1.8329]	Obtained here
	1.83	—	^52^
Guinea	1.11	—	^54^
	1.22	—	^18^
	1.2552	[1.2211, 1.2893]	^26^
	1.4873	[1.4770, 1.4990]	Obtained here
	1.71	[1.44, 2.01]	^52^

**Figure 6:**
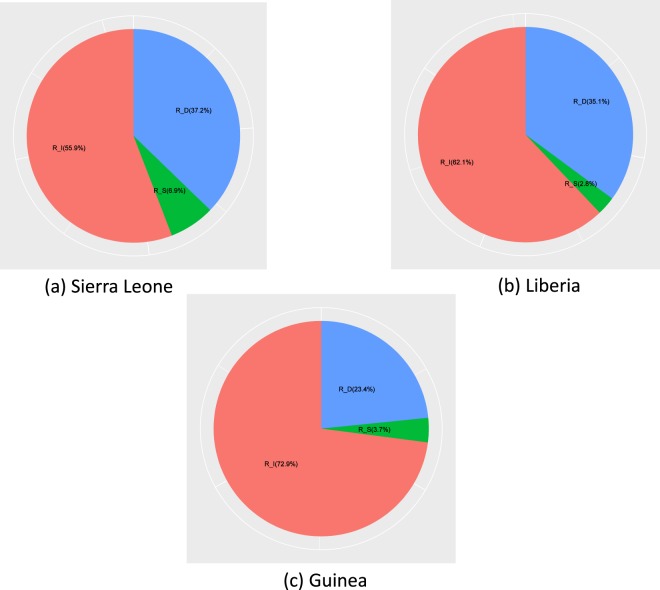
The contribution of infectious component, convalescent component and death component to the basic reproduction numbers *R*_0_. The percentage is displayed with a pie chart.

In order to understand the contribution of related components to *R*_0_ more clearly, we calculated the basic reproduction numbers of either contact with alive infectious transmission or sexual transmission or contact with dead bodies transmission (see Table 1). More precisely, in Sierra Leone, the basic reproduction numbers of infectious component *R*_*I*_ accounted for 0.9331 (55.9%) of *R*_0_, the sexual transmission component *R*_*S*_ accounted for 0.1155(6.9%) and the death component *R*_*D*_ accounted for 0.6218 (37.2%). For Liberia, we obtained 1.1053 (62.1%) for *R*_*I*_, 0.0236 (2.8%) for *R*_*S*_ and 0.6250 (35.1%) for *R*_*D*_. As for Guinea, above three components account for 1.0890 (72.9%), 0.0546 (3.7%), and 0.3493 (23.4%), respectively (see Fig. 6).

We found that the infectious component basic reproduction number was around 1 in the three countries, suggesting the most important factor for the spread of the epidemic is the virus transmission via contact with alive infectious individuals. In addition, we found that the epidemic was spread through contacting dead bodies in the funeral. Thus, we conjecture that the spread of Ebola can be effectively controlled by isolation and proper handling of dead bodies. The value of *R*_*S*_ is less than 10% in three countries, and not enough to cause an outbreak of disease. However, sexual transmission can prolong the outbreak of Ebola.

### Sensitivity Analysis and Disease Control

It is well known that the basic reproduction number (*R*_0_) is a very important parameter in the infectious disease model, which determines whether the could spread. In our model, *R*_0_ is determined by the parameters of *γ*, *θ*, *η*, *ρ*, *p*, *β*_1_, *β*_2_, *β*_3_ and *δ*. In order to identify the impacts of thses parameters on Ebola transmission and prevalence, we used the Latin hypercube sampling method and partial rank correlation coefficient (PRCC)^39^. Using model (1), 2000 samples are randomly generated by assuming a uniform distribution for each parameter based on values from Table 1.

**Figure 7:**
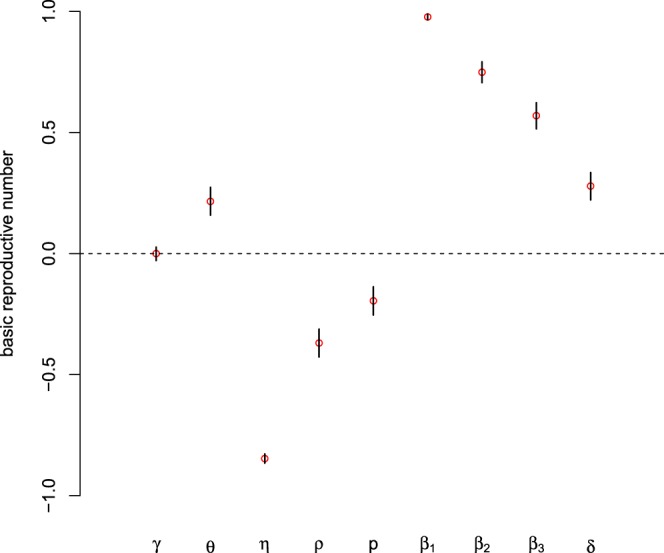
The Partial rank correlation coefficient values for model (1). The basic reproduction number (*R*_0_) was used as the response function.

We choose parameters of interest as the input variables, and the value of *R*_0_ as the output variable. The PRCC values of eight parameters and corresponding p-values are computed. Given the common ways of controlling measures in three countries, and to avoid repeated work, here, we only presant Sierra Leone’s sensitivity analysis. The results of Sierra Leone are illustrated in Table 3 and shown in Fig. 7. The larger the absolute value of PRCC, the stronger the correlation between the input parameters and *R*_0_. From Table 3, it is evident that *η*, *ρ* and *p* have negative impact upon *R*_0_, while *γ*,*θ*, *β*_1_, *β*_2_, *β*_3_ and *δ* have positive impact. Furthermore, Fig. 7 shows that contact with alive infectious transmission rate *β*_1_, infectious class’s removal rate *η*, sexual transmission rate *β*_2_ and contact with dead bodies transmission rate *β*_3_ are the most sensitive parameters on *R*_0_.

**Table 3:** Partial rank correlation coefficients (PRCCs) for *R*_0_ and each input parameter.

Input parameter	PRCC	95% Confidence Interval	*p* value
*γ*	0.0001	[−0.0291, 0.0264]	0.8413
*θ*	0.2152	[0.1584, 0.2733]	0.0015
*η*	−0.8468	[−0.8655, −0.8278]	<0.0001
*ρ*	−0.3695	[−0.4272, −0.3122]	<0.0001
*p*	−0.1953	[−0.2540, −0.1375]	0.0021
*β*_1_	0.9769	[0.9648, 0.9888]	<0.0001
*β*_2_	0.7486	[0.7056, 0.7916]	<0.0001
*β*_3_	0.5692	[0.6227, 0.5148]	<0.0001
*δ*	0.2785	[0.3343, 0.2212]	<0.0001

**Figure 8:**
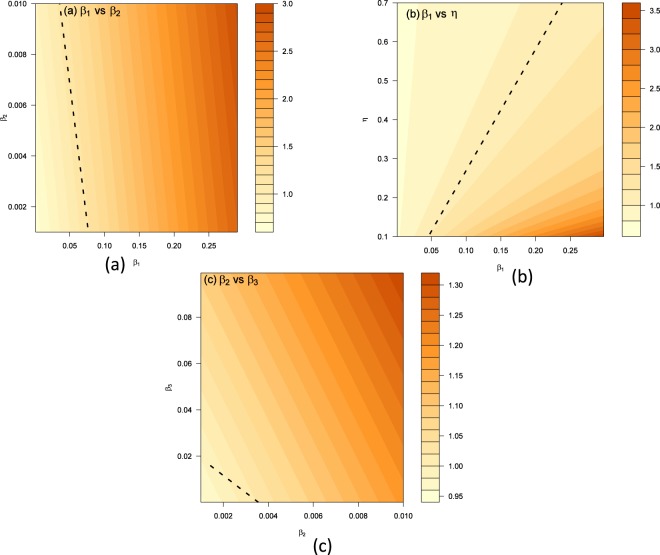
The contour plot of *β*_1_, *η*, *β*_2_ and *β*_3_ on the basic reproduction number *R*_0_. The black dashed curve is the contour of *R*_0_ = 1.

Through the result of sensitivity analysis, we focus on quantifying the effects of *β*_1_, *η*, *β*_2_, and *β*_3_ on the basic reproduction number. In the numerical simulations, we choose *γ* = 1/9.31, *ρ* = 1/2, *p* = 1/87.35, *θ* = 0.69, *δ* = 0.9511, and vary the four controllable parameters from 0.001 to 0.3 for *β*_1_, from 0.001 to 0.01 for *β*_2_, from 0.1 to 0.7 for *η* and from 0.0001 to 0.1 for *β*_3_. Fixing values of *η* and *β*_3_, *β*_2_ and *β*_3_, *β*_1_ and *η* simultaneously, we plot the contour figure of *R*_0_ in terms of *β*_1_ and *β*_2_, *β*_1_ and *η*, *β*_2_ and *β*_3_ are displayed in Fig. 8, to explain the specific value of controllable parameters when the basic reproduction number can be brought below 1.

From Fig. 8(a,b), we find that it is possible to reduce the basic reproduction number to be below 1 with a certain range of the parameters, which means reducing the rate of contacting with infectious individuals may be an effective method to control the outbreak of Ebola. In Fig. 8(c), we show the influences of *β*_2_ and *β*_3_ on the basic reproduction number. We can see that even if we can implement proper handling of dead bodies to reduce *β*_2_, the disease is hard to be controled with a high sexual transmission outbreaks.

## Conclusion and Discussion

Ebola has a high mortality rate and it has caused in many West Africa countries, especially in Sierra Leone, Liberia and Guinea in 2014, which led to many deaths. In this paper, we developed a mathematical model to explore the transmission dynamics of Ebola virus. As a comprehensive consideration of the transmission of the virus, the model includes the effect of contact with infections, contact with dead bodies, and sexual contact with convalescent survivors. Furthermore, we qualitatively analyzed the impact of sexual transmission on the prevention and control of the Ebola. By fitting the model to the cumulative numbers of infected cases (Fig. 4), we obtained some estimates of the parameters (Table 1).

Based on reasonable parameter values and the existing literature, we calculated the basic reproduction number *R*_0_ and 95% confidence interval for Sierra Leone, Liberia and Guinea as 1.67 (95%CI:1.59–1.76), 1.82 (95%CI:1.77–1.83) and 1.49 (95%CI:1.48–1.50), respectively. This indicates that Liberia is the most seriously affected country among these three countries by the end of October 25, 2014. Our results seem to be in accordance with previous work carried out in this field (Table 2).

To be more precise, we consider the contribution of different settings for transmission of Ebola to *R*_0_ with no effective control measures. In our results, the ranking proportional values of *R*_*I*_, *R*_*S*_, and *R*_*D*_ accounting for the basic reproduction number was the same in the three countries, the biggest proportional value among the three countries is *R*_*I*_, then *R*_*D*_, and the smallest is *R*_*S*_. The results imply that *R*_*I*_ and *R*_*D*_ transmission routes play more crucial roles in the Ebola transmission in Sierra Leone, Liberia and Guinea. Similar components of *R*_0_ were identified by Barbarossa estimated *R*_*C*_ = 0.25 (0.16, 0.29), *R*_*H*_ = 0.15 (0.04, 0.23), and *R*_*F*_ = 1.05 (0.48, 1.49)^27^, but in our study, the value of *R*_*I*_ is the largest, which may due to the fact that *R*_*I*_ includes both the infections generated in the community *R*_*C*_ and that in the hospital *R*_*H*_ in their model. However, contribution of sexual transmission to overall *R*_0_ is 0.12% in^16^.

Sensitivity analysis is vital to identify key parameters and find effective control strategies for combatting the spread of the disease. The result of sensitivity analysis indicates that contact with alive infectious transmission rate *β*_1_, infectious class’s removal rate *η*, sexual transmission rate *β*_2_ and contact with dead bodies transmission rate *β*_3_ are the most sensitive parameters to *R*_0_ than any other parameters. The simulation results show *β*_1_ imacts the most of the value of *R*_0_, which indicates that protective among the community and medical workers should be strengthened, and measures to isolate infected people in turn reduce the rate of infection in the population. Remarkably, the sexual transmission component *R*_*S*_ accounted for 6.9%, 2.8% and 3.7% in Sierra Leone, Liberia and Guinea respectively. However, the contribution of sexual transmission to overall *R*_0_ is 0.12% in^16^. Our results show that the potential risk of sexual transmission will cause greater difficulties in terms of controlling the spread of EVD.

The knowledge of the potential for sexual transmission has led to the WHO to make recommendations that require recovered men to avoid sexual intercourse and use condoms as much as possible^53^. Moreover, the public health community must be vigilant in responding to these cases. Most of the population in the affected area are still susceptible, and as long as the virus is not eradicated, the risk of an outbreak could still be high.
